# Writing for *JOOT*: raising standards in clinical research and evidence synthesis

**DOI:** 10.1186/s10195-025-00868-5

**Published:** 2025-08-04

**Authors:** Filippo Migliorini, Filippo Randelli, Alberto Di Martino, Fabrizio Rivera

**Affiliations:** 1 Department of Trauma and Reconstructive Surgery, University Hospital Halle (Saale), Martin-Luther-Universität Halle-Wittenberg, Halle, Germany; 2 Department of Orthopaedic and Trauma Surgery, Academic Hospital of Bolzano (SABES-ASDAA), Via Lorenz Böhler 5, 39100 Bolzano, Italy; 3 Department of Life Sciences, Health, and Health Professions, Link Campus University, Via del Casale di San Pio V, 00165 Rome, Italy; 4 Hip Department-CAD, ASST Gaetano Pini-CTO, Piazza Cardinale Andrea Ferrari 1, 20122 Milan, Italy; 5 1st Orthopaedic Department, IRCCS – Istituto Ortopedico Rizzoli, Bologna, Italy; 6 Department of Biomedical and Neurimotor Sciences, University of Bologna, Bologna, Italy; 7 Department of Orthopaedics and Traumatology, Ospedale SS Annunziata, ASL CN1, Via Ospedali, 9, 12038 Savigliano, Italy

**Keywords:** Guidelines, Recommendations, Scientific writing, Manuscript preparation, Journal submission, Publication strategy, Research

## Abstract

This editorial aims to guide prospective authors in effectively preparing and structuring a manuscript for submission to *JOOT*. Despite the increasing scientific quality of many submissions, the Editorial Board frequently receives manuscripts that fail to meet fundamental standards in structure, style or adherence to journal requirements, which may compromise their chances of acceptance. Scientific writing is a crucial skill, and tailoring a manuscript to the expectations and guidelines of the target journal is vital for successful publication. This article offers practical recommendations to enhance manuscript preparation, improve clarity and align submissions with the editorial standards of *JOOT*.

## Editorial overview

The *Journal of Orthopaedics and Traumatology* (*JOOT*) is the official scientific publication of the Italian Society of Orthopaedics and Traumatology (SIOT). Founded in 2000 by Prof. Francesco Pipino [1], *JOOT* was established to promote the visibility of Italian orthopaedic research and to foster international academic exchange. Initially published in print format, the journal gained international recognition quickly. By 2006, approximately 30% of the articles were authored by researchers based outside of Italy, a proportion that rose significantly to 70% by 2014. This increase in international engagement marked a pivotal phase in the journal’s development. In 2014, under the leadership of Editor-in-Chief Prof. Marco D’Imporzano and Executive Editor Dr. Luca Pierannunzii, *JOOT* adopted an open-access publication model. This transition was strategically implemented to enhance the journal’s visibility, accessibility and scientific credibility, with the specific objective of attaining an official impact factor. These concerted efforts culminated in the successful assignment of the journal’s first impact factor in 2018. Since then, *JOOT* has experienced substantial growth, establishing itself as a leading international journal in the fields of orthopaedics and traumatology. Its scientific impact has continued to expand, as evidenced by the increasing number of citations and the steady rise of its impact factor, which reached a value of 3.0 in 2024 [2, 3]. The journal has recently strengthened its focus on high-priority scientific topics; in 2024, a thematic collection was launched in the field of regenerative medicine, featuring contributions from globally recognised experts such as Giannoudis [4] and Blokhuis [5]. In response to the increasing volume of submissions, which has more than doubled in recent years, the Editorial Board has undergone a substantial expansion, now comprising over 400 qualified reviewers. Looking ahead, *JOOT* is committed to fostering innovation, scientific excellence and global collaboration. With the sustained dedication of its editorial leadership, reviewers and contributing authors, the journal is well positioned to advance orthopaedic knowledge further and continue serving as a trusted academic platform for future generations of clinicians and researchers.

In this editorial, we aim to guide prospective authors in effectively preparing and structuring a manuscript for submission to *JOOT*. Despite the increasing scientific quality of many submissions, the Editorial Board frequently receives manuscripts that fail to meet fundamental standards in structure, style or adherence to journal requirements, which may compromise their chances of acceptance. Scientific writing is a crucial skill, and tailoring a manuscript to the expectations and guidelines of the target journal is vital for successful publication. This article offers practical recommendations to enhance manuscript preparation, improve clarity and align submissions with the editorial standards of *JOOT*.

## Crafting a compelling introduction

There are no established guidelines governing the rationale composition within the introduction section of scientific manuscripts. It is advised that the introduction be limited to a maximum of 500 words. Sentences should be articulated through clear and concise statements, facilitating coherent transitions to subsequent statements. A minimum of one relevant citation must support each assertion to uphold the rigour of the scientific discourse. Avoid well-known details while ensuring that sufficient background information is provided to orient the readership effectively. A detailed discussion of results from prior investigations should be omitted, with a methodical transition from a broad contextual framework to the specific objectives of the study in question. The introduction should be systematically organised into four sections: background, literature gap, purpose and hypothesis. The background section introduces the research topic broadly, underscoring its social relevance in terms of its social, economic and medical implications. A succinct state-of-the-art overview should be provided, delineating current knowledge and identifying uncertainties or controversial aspects within the field. Given that the intended audience comprises subject matter experts, it is unnecessary to elaborate on fundamental concepts; the focus should be on delivering precise information efficiently and effectively. The literature gap section is a crucial component of the manuscript. It must articulate gaps, limitations or challenges within the existing literature to justify the need for the current research endeavour. This section is paramount as it should convincingly engage the reader regarding the necessity and relevance of the study, highlighting its importance within the scientific community. In the purpose section, it is imperative to articulate the research aim concisely in a clear statement. This establishes the framework for the entire study, elucidating how the research addresses the identified knowledge gap or problem while emphasising the significance and potential impact of the research findings. The outcomes of interest should be explicitly stated alongside the objectives or research questions. The endpoints to evaluate these outcomes must also be declared, accompanied by an evidence-based hypothesis. The scientific article should be structured around this hypothesis, which will subsequently be confirmed or refuted within the results section, followed by a comprehensive examination in the discussion section. It is suggested that the introduction section end with two to three research questions that will be developed in the other sections of the manuscript.

## Presenting a transparent and reproducible methodology

The methods section of a scientific article provides a detailed and transparent explanation of how the study was conducted, thereby assessing validity and reproducibility. Taking into account the research questions, the authors should present a clear methodology according to current standards of reporting. This chapter should be approximately 1000 words long. It is crucial not to report any results. Indeed, a common mistake is including patient demographics or enrolment outcomes in this section. From a scientific perspective, this section is the easiest to write, as it provides a straightforward and detailed account of the methodology employed in the study, adhering to highly detailed guidelines. Understanding the difference between a clinical trial and other studies is crucial for choosing the appropriate guidelines. The World Health Organization (WHO) defines a clinical trial as “any research study that prospectively assigns human participants or groups of humans to one or more health-related interventions to evaluate the effects on health outcomes”. *JOOT* follows the guidelines outlined in the Enhancing the QUAlity and Transparency Of health Research (EQUATOR) network. Clinical trials must adhere to the Consolidated Standards of Reporting Trials (CONSORT) guidelines [6], while observational retrospective investigations must follow the Strengthening the Reporting of Observational Studies in Epidemiology (STROBE) guidelines [7]. Authors are strongly encouraged to cite these guidelines in the text and upload the guideline checklist as supplementary material. Regardless of the study type, both guidelines share similar constructs. Briefly, the time frame of patient enrolment, the design and the institution must be declared first. A statement on the ethics review must include the committee’s name, date, and ID for all research studies considered in *JOOT*. In addition, the investigation must adhere to the principles outlined in the Declaration of Helsinki and its later amendments. Patients must be informed of the nature and purpose of the study and must sign a written consent to participate. Eligibility criteria must be clearly and thoroughly described. The study setting must be clearly defined, including the location and any materials, software or instruments used. Detail the experimental workflow, including data collection, interventions, or testing methods. If applicable, divide this section into subheadings for clarity and add figures or tables. All instruments, software and third-party materials must be declared, including their names, manufacturers and related cities and states. Clearly define primary and secondary outcomes, mentioning the criteria or definitions used to assess these outcomes and, if applicable, the follow-up. Software, implants and products should be reported using their commercial names, with the names of manufacturers, cities and states included in parentheses. The systematic review shall strictly follow the Preferred Reporting Items for Systematic Reviews and Meta-Analyses (PRISMA) guidelines [8]. The literature search shall be updated and not older than 3 months before submission. The Population, Intervention, Comparison, and Outcome (PICO) framework shall be used and reported. Three databases shall be used for the literature search. Authors shall report the date of the literature search and the filter used. The authors shall also report all strings and MEdical Subject Headings (MESH) used for the database search. Database search and data extraction shall be conducted independently by two reviewers. The quality of the methodology shall be accurately explored. *JOOT* invites authors to follow the Cochrane Handbook for Systematic Reviews of Interventions (The Nordic Cochrane Collaboration, Copenhagen) [9]. The Risk of Bias in Non-Randomised Studies-of Interventions (ROBINS-I) shall be used for non-randomised controlled trials, and the Risk of Bias tool for randomised controlled trials. Authors shall state the level of evidence of each included study, according to the Oxford Centre for Evidence-Based Medicine (OCEBM) [10].

## Structuring and reporting the results

Results must adhere to the specified guidelines and be approximately 500–750 words long. Original studies should systematically categorise results into three primary sections: patient recruitment, patient demographics and results synthesis. In the patient recruitment section, authors must provide a comprehensive account of the number of patients included and excluded at each study stage, elucidating the rationale for exclusions. Furthermore, the authors must present a figure detailing the flowchart of the patient enrolment process. The subsequent section should outline the general characteristics of patient demographics, including a table for reporting these demographics, which is strongly encouraged. Please carefully choose the figures and tables to accompany the text; moreover, avoid repeating the same sentence and concept in the main text and legends. For comparative studies, assessing baseline comparability is critical to ensure reliability. The concluding section should present the synthesis of the results, organised according to the research questions posed in the introduction section. Systematic reviews should comprise four fundamental sections: search results, methodological quality assessment, patient demographics and results synthesis. Within the search results, authors must provide a comprehensive explanation of the number of articles included and excluded at each phase of the review, clearly articulating the reasons for exclusion. In addition, authors should incorporate a figure that illustrates the flowchart of the search results. In the patient demographics section, authors are expected to discuss the demographics of the patients, as well as the general attributes of the included studies. Including a detailed table to present this information is encouraged. It is essential that authors appropriately cite all studies included in this section. The final section will articulate the synthesis of the results. If a meta-analysis is undertaken, a funnel plot will be necessary, with any observed asymmetry assessed using Egger’s or Begg’s tests. Furthermore, the forest plot should be reported to enhance transparency.

## Developing a critical and insightful discussion

This chapter will begin by presenting the main findings of the study and discussing whether these findings support or refute the hypothesis. Discussions should be approximately 1000 words long. There are no formal requirements for writing the discussion section; however, authors might find it easier to start the introduction by summarising the main findings of the study; then, the limits and strengths of the study should be analysed. These limitations should be discussed extensively, as they are crucial for transparency. Acknowledging limitations demonstrates that authors are aware of potential weaknesses in their work, enhances confidence in their scientific rigour, aids readers in accurately interpreting the results and prevents incorrect or overly generalised conclusions. Finally, one paragraph is developed for each research question, comparing the study findings with the best available literature on the topic. Finally, the clinical relevance of their findings, potential mechanisms, and implications for clinicians or policymakers should be thoroughly analysed and reported. This chapter will conclude by addressing unanswered questions and suggesting directions for future research.

## Data Availability

All data are contained within the manuscript.
